# CRISPR Screens in Synthetic Lethality and Combinatorial Therapies for Cancer

**DOI:** 10.3390/cancers13071591

**Published:** 2021-03-30

**Authors:** Laia Castells-Roca, Eudald Tejero, Benjamín Rodríguez-Santiago, Jordi Surrallés

**Affiliations:** 1 Genome Instability and DNA Repair Syndromes Group, Sant Pau Biomedical Research Institute (IIB Sant Pau) and Join Unit UAB-IR Sant Pau on Genomic Medicine, 08041 Barcelona, Spain; 2 Genetics Department, Hospital de la Santa Creu i Sant Pau, 08041 Barcelona, Spain; brodriguezs@santpau.cat; 3 Genetics and Microbiology Department, Universitat Autònoma de Barcelona, 08193 Bellaterra, Spain; 4 Sant Pau Biomedical Research Institute (IIB Sant Pau), 08041 Barcelona, Spain; ETejero@santpau.cat; 5 Center for Biomedical Network Research on Rare Diseases (CIBERER) and Sant Pau Biomedical Research Institute (IIB Sant Pau), 08041 Barcelona, Spain

**Keywords:** CRISPR screen, synthetic lethality, combinatorial therapy, cancer therapeutic resistance

## Abstract

**Simple Summary:**

The synthetic lethality (SL) clinical success of PARP inhibitors in homologous recombinant deficient tumors has established a new concept for cancer treatment. For decades, efforts have centered on identifying genetic interactions for determining essential tumoral genes, and more recently, SL interactions to determine combinational treatments against persistent cancer reappearance. Currently, the feasibility of CRISPR screen methodology has emerged as the state of the art for uncovering new SL or viable interactors in the biology and treatment of cancers. We present the up-to-date research of numerous laboratories that take advantage of the genome-wide forward genetic CRISPR screen tools and protocols to identify cancer biomarkers, genetic interactions and novel therapies. Indeed, investigations are nowadays focused on defining innovative combinatorial treatments based on SL interactions. By coupling different drugs, concentration treatments can be lowered and therefore toxicity reduced. CRISPR screen technologies have deeply impacted cancer research to promote a robust advance in combined therapies.

**Abstract:**

Cancer is a complex disease resulting from the accumulation of genetic dysfunctions. Tumor heterogeneity causes the molecular variety that divergently controls responses to chemotherapy, leading to the recurrent problem of cancer reappearance. For many decades, efforts have focused on identifying essential tumoral genes and cancer driver mutations. More recently, prompted by the clinical success of the synthetic lethality (SL)-based therapy of the PARP inhibitors in homologous recombinant deficient tumors, scientists have centered their novel research on SL interactions (SLI). The state of the art to find new genetic interactions are currently large-scale forward genetic CRISPR screens. CRISPR technology has rapidly evolved to be a common tool in the vast majority of laboratories, as tools to implement CRISPR screen protocols are available to all researchers. Taking advantage of SLI, combinatorial therapies have become the ultimate model to treat cancer with lower toxicity, and therefore better efficiency. This review explores the CRISPR screen methodology, integrates the up-to-date published findings on CRISPR screens in the cancer field and proposes future directions to uncover cancer regulation and individual responses to chemotherapy.

## 1. Introduction

After advances in genome sequencing, maximum interest has been placed on interpreting the genetic code [1] and understanding how genetic alterations cause cancer and diseases, aiming to efficiently translate available biological data into feasible treatments. Indeed, tumor heterogeneity, resulting from the accumulation of genetic dysfunctions, reveals the complex molecular diversity that regulates therapeutic responses. Novel combinatorial therapies and personalized medicine approaches, to reduce drug resistance and cancer reappearance, have been set as key for the highly expected translational medicine developments in the 21st century. The toolkit to effectively address this issue has become mammalian genome editing by the use of Clustered Regularly Interspaced Short Palindromic Repeats (CRISPR)/CRISPR-associated protein 9 (Cas9) system. The CRISPR pathway functions as an adaptive immune system in prokaryotes and archaea [2,3], but its design simplicity, efficiency, achievable cost and multiplex editing possibilities have prompted its applications to widely spread among the research community and to exponentially rise in the scientific literature, contrary to the usage of other nuclease enzymes [4,5,6,7,8,9,10].

CRISPR systems have lately been classified as Class 1, comprehending Type I, III and IV, and Class 2 with Type II, V and VI, reviewed in [11]. Interestingly, newly discovered Class 1 and 2 variants are predicted to have divergent roles from adaptive immunity, especially several gene families associated with type III systems that are involved in signal transduction and regulatory functions, as reviewed in [12]. In 2012, Jinek et al. demonstrated that the Type II (from Class 2) endonuclease Cas9 could be programmed with single RNA molecules to cleave specific DNA sites for genome targeting and editing [13]. Only two years after, some laboratories successfully designed, engineered and applied their own libraries and protocols for large-scale forward genetic screens in human [14,15,16] and mouse [17] cell lines. CRISPR wide-genome screens have revolutionized loss-of-function screens, until then performed using RNA interference (RNAi) or short hairpin RNA (shRNA). The CRISPR technology improves transcription suppression and reduces off-target efficiency in RNAi screens, while simultaneously increasing sensitivity to score for essential genes [18]. Undoubtedly, CRISPR knockout (CRISPRko) screens are a highly powerful technique when combined with biocomputational methods to elucidate essential cancer genes [19,20,21], which are crucial to both determine cell growth and division for disease progression, and also to define important therapeutic targets.

Employing a catalytically inactive Cas9 (dCas9) fused to different functional domains allows competent transcriptional activation or repression [22]. Indeed, dCas9 by itself represses gene transcription by directly blocking RNA polymerase and this repression is enhanced by the fusion of dCas9 with the Krüppel-associated box (KRAB) domain. A CRISPR interference (CRISPRi) library using this system is already available [23] and it is useful to discern among multiple transcript isoforms [24] or cis-regulatory elements [25]; however the gene repression level is dependent on each sequence [26] and the transcriptional start site (TSS) location of each gene [23,27]. Divergently, CRISPR activation (CRISPRa) second generation libraries are based on the fusion of the herpes virus tetrameric transcription activation domain VP64 with the SunTag signal amplification system [28] or with a sgRNA containing two RNA hairpin aptamers recognized by the MS2 bacteriophage coat protein and a trimeric fusion protein complex (VP64, p65 and HSF1) to make a synergistic activation mediator (SAM) [29,30], among others. CRISPRa libraries offer the possibility to implement gain-of-function screens, formerly restricted to overexpression of costly cDNA libraries, with overexpression beyond the physiological levels and endogenous regulation [30].

The continuous expansion of CRISPR/Cas technology enables genetic modifications not only at a genomic *loci* level, but also at the expression regulation (epigenetic and transcriptional) one. Currently, numerous laboratories have taken the advantage of this methodology’s simplicity to design and analyse screens in order to find innovative leads to treat the most complex disease of all, cancer. Here, we provide a technical overview of the protocol steps to perform genome-wide forward genetic CRISPR screens in cancer cell lines and we review recent approaches using this methodology for cancer. Efforts are focused on unravelling genetic interactions (GI) in vitro and in vivo, to find novel synthetic lethal (SL) or viable interactor genes that potentially result in combinatorial therapeutic targets, synergistically or antagonistically, with known drugs.

## 2. CRISPR/Cas9 Screens Steps

**Figure 1:**
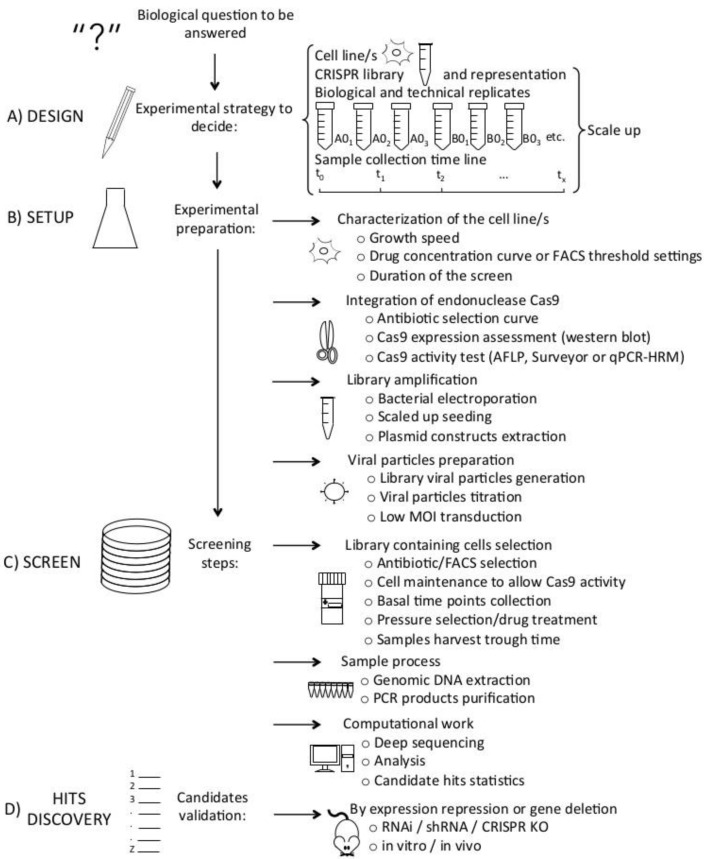
Schematic outline of CRISPR/Cas genome-wide screens. (**A**) Previous to the screen, the experimental design must fit the biological question to solve. The experimental strategy includes selection of cell line/s, CRISPR library and its representation, number of biological and technical replicates and the total amount of samples to harvest, all together will dictate cell quantity and number of plates to culture. (**B**) Before starting the actual experiment, a setup preparation takes place: characterization of the cell line/s, integration of the endonuclease Cas9, library amplification, viral particles preparation and cell transduction. (**C**) The screening steps are: selection of the transduced cells, collection of the basal samples, drug treatment or pressure selection, samples harvest, DNA extraction, PCR amplification, NGS, computational analysis and candidate compilation. (**D**) For candidate hits validation, gene expression may be repressed by RNAi or shRNA or genes may be deleted using CRISPR KO, in vitro and in vivo.

CRISPR/Cas9 screens are powerful genomic tools to elucidate the mechanistic aspects of cancer and to define biomarkers and therapies. Despite being challenging, the design and execution of CRISPR pooled screens have become available to the vast majority of laboratories. Scientists merely need to adjust the appropriate experimental strategy to the biological question to be solved. A schematic overview of the general steps is shown in Figure 1. The engineering of a cell population is necessary for high-throughput screening in order to alter gene expression and define gene functions. Accordingly, the CRISPR/Cas system must be introduced to every cell, which should potentially have one genomic *locus* modification, hence each cell must receive a single sgRNA.

### 2.1. CRISPR Library

The type of CRISPR library must adjust to the biological question to be answered: CRISPRko libraries eliminate gene expression for loss-of-function or SL/viable interactions screens, CRISPRa libraries to overexpress genes for gain-of-function screens and CRISPRi libraries to block TSS and investigate gene repression. The number and diversity of gene knockouts (KO) or genomic region modifications depends on the approach to be taken: when aiming to find any GI in the genome, a CRISPR library will contain ~20,000 targeting molecules; alternatively, if the screen focuses on a smaller fraction of genes with related function, for instance: transcription factors [31], nuclear proteins, kinases [15], etc., libraries will target only hundreds of genes. Consequently, subpooled libraries allow screens with reduced numbers of cells and reagents.

sgRNAs selection for the CRISPR library must be done by cutting efficiency (which varies among sgRNAs and sequence context) and target specificity (as several mismatches along the sgRNA can be tolerated causing off-target mutations). Recently, many computational models and resources offer further advances in efficiency and specificity prediction for the design and selection of CRISPR pooled libraries [32,33,34,35]. sgRNA libraries are composed of a set of oligonucleotides, ~20 nt sequences targeting genes or non-coding regions (promoter elements, putative regulatory sequences). However, truncated sgRNAs (17–18 nt) have been proposed to enhance on-target activity in CRISPRko screens [36]. Synthetized pooled CRISPR libraries are already available to scientists at addgene.org to screen for many species; for gene KO or *loci* activation or inhibition; in a large-genome scale or as subpools; and by way of plasmids or viral preparations. Additionally, alternative options may be obtained from commercial companies. Remarkably, selected oligonucleotides are designed to be redundant. Distinct molecules target the same genomic sequence to compensate for effects of sgRNA diverse efficiency and off-targets consequences. It is recommended to use at least four independent sgRNAs for each genome sequence targeted [15], additional guides increase up to 5% more candidate hits in the analysis [37]. For CRISPRko libraries, algorithms were created to determine sgRNA target sequences directed to 5′ exons and to lower off-targets [14,15], to increase target efficiency [15,38] and optimize sgRNA [39,40]. For CRISPRa libraries, sgRNAs are targeting sequences upstream TSS and for CRISPRi, downstream TSS [30]. In most of the cases, the oligonucleotides are cloned into a lentiviral plasmid that is used for the generation of viral particles, as lentivirus transduce dividing and non-dividing cells, integrate into the genome and have a larger insert size capacity compared to adeno-associated virus. A vial of the amplified plasmidic library used to screen could be kept for deep sequencing, when the final readout will be compared to assess library representation.

### 2.2. Considerations for Screening in Cancer Cell Lines

Together with the choice of the library comes the selection of an appropriate cell line. When possible, the best is to perform the screen in multiple cell lines [15,38], in order to reduce the differences in growth, transduction, background mutations or sensitivity to the therapy to test. In general, mammalian cell lines are diploid, but most cancer cell lines are genetically instable, thus may be polyploid for multiple *loci*. Since the efficiency of sequence alterations depends on the targeted number of copies, the CRISPR/Cas system may unevenly engineer each genetic *locus* in genetically instable cancer cell lines. Accordingly, candidate hits of a polyploid cell line conducted screen will result in worse quality compared to a diploid or haploid cell line [15,38].

CRISPRko screens promote small insertions or deletions in the specific *loci* by repairing the generated DSBs through non-homologous end joining (NHEJ). This repair pathway will be employed by many cancer cell lines, where the homologous direct repair (HDR) is impaired [41]. Nevertheless, NHEJ is the preferred pathway to repair DSB, in G1 as in G2, and the HDR is only used in ~15% [42]. Moreover, genome-wide screens are also viable in NHEJ deficient cells, since this repair pathway is fully compensated by the alternative end joining (alt-EJ) route [43].

### 2.3. Setting Up the Experimental Protocol

Once appropriate cell lines and a library are chosen, the experimental strategy must be designed. At this moment, both biological and technical replicates, together with the number of samples to be collected over time (with the corresponding baseline time points or controls) may be established. Besides, library representation must be set to ensure a sufficient diversity representation of sgRNAs in the cell population, 500–1000 cells per sgRNA are recommended [44]. All these parameters will determine the scaling up of the experiment: the initial number of cells, total days in culture and the amount of harvested cells for each time point (~30 million). Thus, between 80 and 200 million cells should be transduced to screen [14,15,38], cultured in many tens of plates and maintained/passed for weeks, being thus indeed a large-scale procedure.

Preceding the actual experiment, several setup steps must be done, as settings may vary depending on each specific cell type. First, the characterization of the cellular model: doubling time, drug concentration or fluorescence activated cell sorting (FACS) threshold selection settings, duration of the screen, etc. Secondly, it must be decided if the endonuclease Cas9 will be delivered together with the sgRNAs, increasing plasmid size at the cost of reducing the transduction efficiency, or if Cas9 will be already stably expressed by the cell population [14,45], requiring longer selection steps. When the chosen cell type allows clonal expansion, the best Cas9 expressing cells are easily selected, hence, an antibiotic concentration curve should be previously prepared. Next, Cas9 activity must be tested by introducing different sgRNA constructs and evaluating the cutting efficiency via conventional amplified fragment length polymorphism (AFLP), SURVEYOR assay or quantitative PCR high-resolution melting (qPCR-HRM) curve analysis technique [46]. Once the cell population for the screen is engineered, it will be transduced by the library viral particles at a low multiplicity of infection (MOI), around 30% more of cells than viral particles [14,15,38], to enrich for cells that integrate a single sgRNA cassette. Therefore, the third optimization step is to titrate the amount of viral particles required to transduce the selected cell line in the ranked low MOI, which directly depends on the chosen cell line and the viral particles batch.

**Figure 2:**
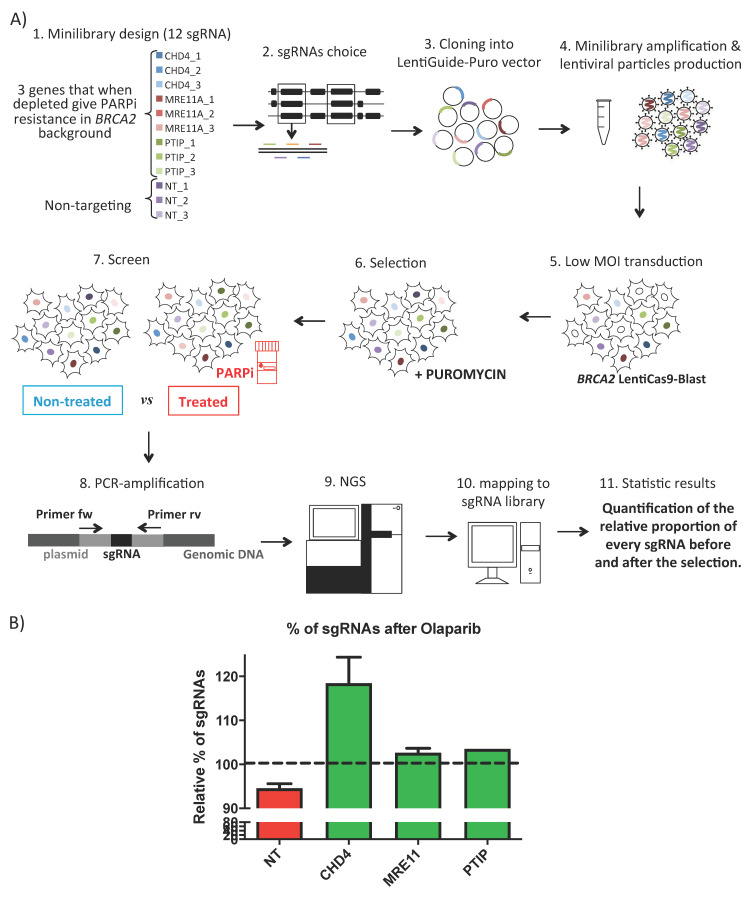
(**A**) Proof of principle CRISPR/Cas screen. 1. Minilibrary design with 3 sgRNAs for each gene of interest (3 genes that when depleted give PARPi resistance in *BRCA2* background) plus 3 NT sgRNAs. 2. sgRNAs choice (at 5′ common exons, with minimal off-targets). 3. sgRNAs cloning into LentiGuide-Puro vector. 4. Minilibrary constructs amplification and lentiviral particles production. 5. Low MOI transduction into *BRCA2* fibroblast cells previously transduced with the plasmid LentiCas9-Blast. 6. Puromycin selection of cells with transduced sgRNAs, during 9 days. 7. Screen: non-treated and treated samples collection, after 7 days of 100 nM olaparib. 8. PCR of the sgRNA region. 9. NGS by MiSeq. 10. FASTQ files analysis and mapping to sgRNA library. 11. Statistic significant candidate hits by quantification of the relative proportion of every sgRNA before and after the selection. (**B**) Relative percentage of sgRNAs in olaparib treated samples versus non-treated. Average of the three-targeted genes and the NT sgRNAs. Only sgRNAs with significant *p*-values from the student t-test statistics are shown. Values: CHD4 118.4%, 2 sgRNAs; MRE11A 102.6%, 3 sgRNAs, PTIP 103.5%, 1 sgRNA and NT 94.5% data from four sgRNA with no significant *p*-values (CHD4_3, PTIP_1, PTIP_3 and NT_2).

To prove that the screen settings will work, a proof of principle experiment is recommended. An example targeting genes (MRE11, CHD4 and PTIP) [47] that when mutated induce resistance to the PARP inhibitor (PARPi) olaparib in the *BRCA2* background is shown in Figure 2. (The data presented in this study are available on supplementary (Table S1) information).

### 2.4. CRISPR Screen

In vitro CRISPR screens with immortalized cell lines have generally no limitation in cell number, thus wide-genome screens using millions of cells are easily feasible, while screens in primary cell lines, organoids or in vivo are commonly limited in cell number, thus more directed screens to particular gene families, biochemical routes or protein types with subpooled libraries are more suitable.

Following transduction, cell selection may be done by antibiotic resistance or FACS. Several days of culture are necessary for the sgRNA-Cas9 duplex to alter the genome, cells must be kept in antibiotic selection to screen during 7 to 14 days [14,15,30,38], to generate KO genes; and for transcriptional upregulation via SAM complex only 4 days [30]. After this period, the screen proceeds with the selected conditions. Cells may be harvested over time in independent replicates, the second replicate enlarges the percentage of hits by 9 to 14% and the third by only less than 5% [37].

At the end the different collected time points will be compared to the baseline time points. Next, genomic DNA is extracted and the integrated sgRNAs amplified by PCR and sequenced, in order to assess the relative abundance of each targeted gene. Overall, the screens can either readout for negative selection or positive selection. Negative selection screens will sensitize cells to the condition and identify essential genes to survive the selective pressure. Thus, the aim is to find sgRNAs less abundant compared to baselines or controls. Conversely, positive selection screens identify genes whose mutation serves as a positive proliferative advantage over the applied condition, offering resistance to the given drug or used stress, hence the readout are sgRNAs with higher abundance than basal time points.

There are two forms of screening process: arrayed and pooled screens. The protocol described above summarizes pooled screens, whereas arrayed screens are performed into multi-well plates and individual reagents must be dispensed into single wells. Phenotypes can be assessed manually, but the readout can be automatized by direct imaging of cells [48], luminescence [49], fluorescence [50,51] or RNA sequencing [52].

### 2.5. Screen Analysis

Genomic DNA is isolated from the biological or technical replicates of harvested samples at different time points. As collected samples are frozen in tens of millions cells, standard procedures must be scaled up. Lentiviral integration of sgRNA cassettes facilitates the analysis by PCR amplification using universal primers that target the common template sequences flanking each specific 20 nt sgRNA. Multiple PCR reactions until expending the sample produce a library of short and highly diverse DNA fragments containing the sgRNA sequences. Every fragment library corresponds to a particular collected time point or replicate and may be used as input for next-generation sequencing (NGS) to obtain sequence reads. Adding diverse sequence barcodes (indexes) to the different libraries/samples allows pooling of many samples for simultaneously sequencing in a NGS equipment. Barcodes are afterwards used to demultiplex information and create individual sample sequence files. Current sequencers are able to provide >10,000 reads per sgRNA [30]. Bioinformatic analysis comparing the total number of reads for every sgRNA between samples and controls after screening conditions are used to obtain candidate genes related to specific sgRNAs. Several algorithms have been designed for this purpose: RIGER [53], RSA [54], STARS [39], castle [55], MAGeCK [56], MAGeCKFlute [57] CRISPRAnalyseR [58], CaRpools [59] among others [60]. Candidate selection or prioritization using different statistical methods together with a gene enrichment analysis [61] are highly recommended. Most of the suitable software packages allow identification of pathways of interest. In addition, both performing multiple tests by comparing each control sample with all the treated replicates and filtering candidate hits by number of comparisons in which sgRNAs show significant *p*-values help to establish a ranked candidate hit list. Alternatively, for arrayed-based screens or read out by FACS studies, candidate hits are found by determining the sgRNAs that produce significant changes in the expression of stained or fluorescent proteins.

### 2.6. Candidate Hits Validation

Promising gene candidates must be validated by means of transcriptional repression, protein inhibition or KO generation, to avoid false positive results. Commonly, hits are analysed together with members of the same pathway, complex or direct upstream and downstream interactors [62,63,64]. Thus, inactivating or repressing a whole functional route reliably proves the rationality of the hit. Definitely, genetic complementation rescue is the ultimately excellent evidence. Since some GI are dependent on the cell line, it is vastly advisable to employ several cell lines in this step, particularly with the usage of genetically heterogeneous tumoral cell lines [65]. Indeed, some authors executed an extraordinary effort validating hit candidates in vitro and in vivo [66,67,68,69,70].

## 3. CRISPR/Cas Screens in Cancer

Medical research aims to identify essential genes and cancer driver mutations. Computational efforts have integrated 501 RNAi screens in numerous well-characterized and heterogeneous cancer cell lines to produce a cancer dependencies map [65]. The Project Score database permits to examine the fitness of 18,009 genes surveyed from 323 cancer cell models, allowing users to interactively select for a particular gene, cancer cell model or tissue type, and rank drug targets, via suitability of genetic biomarkers, clinical datasets or drug development [71]. In the same direction, genome-wide CRISPR-Cas9 essentiality screens on 342 cancer cell lines were combined considering copy number-specific effect [72] and algorithms to correct for on-target and off-target efficiency that have also optimized genome-wide libraries: human Avana and mouse Asiago libraries [39].

Nevertheless, with the goal of defining essential genes, Dede et al. [73] analyzed monogenic sgRNAs CRISPR screens for 684 cancer cell lines, they found that from the 7000 constitutively expressed genes only half were identified as essential by CRISPRko screens and these absent hits were vastly enriched for paralogs. They synchronously targeted multiple genes using Cas12a that processes polycistronic mRNA and facilitates multiplexing of sgRNAs, hence to test particular gene pairs, however on-target efficiency must be still determined. Fortin et al. [74] explored multi-target sgRNAs biases from analysis of CRISPR screens on 391 cancer cell lines, they concluded that sgRNA activities are specific for each cell line, GI as well as one mismatch tolerant sgRNAs may change the evaluation of essential gene hits, and that single nucleotide polymorphisms located in protospacer sequences impair on-target activity.

### 3.1. Synthetic Lethal Screens

Synthetic lethal (SL) interactions (SLI) result in reduced cell survival and they occur from the inactivation of certain gene pairs; although when single, each gene loss still allows cell viability [75]. SL therapy has burst onto the scene as a new successful concept for cancer treatment. The paradigmatic example of SL is the inhibition of poly(ADP-ribose) polymerase (PARP) proteins in homologous recombination (HR) deficient cancer cells, exploding the inherent chromosomal instability of these type of cells [76,77]. Since PARP proteins together with the components of the HR pathway are constituents of proficient DNA repair of DSB and frequently mutated in cancers. PARPi have been proposed to “trap” PARP-1 protein at sites of DNA damage [78,79], inducing a stable interaction between DNA chromatin and PARP-1. These complexes interfere with DNA replication by destabilizing replication forks, conducting to genomic instability and cell death [80,81]. Relevant clinical results with the application of PARPi [82,83,84,85,86] together with the problem of tumoral cells resistance to chemotherapy prompted scientists to search for novel SLI to identify tumor suppressor genes frequently mutated in cancer cells. As HR is commonly attenuated in cancer, many laboratories have focused on fronting PARPi therapy resistances [87]. In order to determine the nature of these non-covalent protein-DNA adducts, Zimmermann et al. [88] conducted three CRISPR screens using human papilloma virus-induced cervical adenocarcinoma (HeLa), retinal pigment epithelium (RPE1-hTERT immortalized) and triple-negative breast cancer–TNBC—with a hemizygous BRCA1 mutation (SUM149PT) with the TKOv1 library [38], showing 73 genes that when mutated cause enhanced olaparib sensitivity, including RNase H2 coding genes of the ribonucleotide excision repair (RER) pathway. Hewitt et al. [89] studied the role for the nucleosome remodelling ALC1 protein by performing CRISPR screens in eHAP iCAS9 expressing non-targeting or the nucleosome remodelling ALC1 sgRNA with the Brunello library [39] and treating them with olaparib. They found that ALC1 produces downstream base excision repair (BER) and its loss generates toxic BER intermediates that result in single-strand gap formation and replication fork collapse, sensitizing cells to PARPi.

SLI have been also defined among paralog genes, through the loss of the second functionally redundant paralog gene. shRNA and RNAi screens have already defined tumoral survival dependent on certain paralogs: SMARCA4 and SMARCA2 either of each are catalytic subunits of the SWI/SNF chromatin remodelling complex [90], or the frequently mutated tumor suppressor ARID1A and its paralog ARID1B, mutually exclusive parts of the SWI/SNF complex [91] or *UBB* and its paralog *UBC* often mutated in ovarian and uterine tumors [65]. Employing shRNA and CRISPR screens on more than six hundred cancer cell lines for each method, Viswanathan et al. [92] observed that the splicing-dependent exon junction complex MAGOH is SL with the MAGOHB-IPO13 axis. Using RNAi and CRISPR screens to identify targets associated with loss of tumor suppressor genes SLI was found between the endosomal sorting complexes required for transport (ESCRT) ATPases VPS4A and VPS4B, which may be therapeutic targets for cancers with 18q or 16q loss [93]. To probe genetic SLI of the inactivated tumor suppressor STAG2 cohesin subunit van der Lelij et al. [94] screened the chronic myelogenous leukemia KBM-7 cells with the Vienna library and they confirmed its paralog STAG1 as the best hit and they defined STAG1 and RAD21 as potential therapies.

Combinatorial treatments are settled as central new therapies to treat tumors, since tumors are context dependent, they may respond differently depending on the tissue affected and the individual health, age, gender or genetic background. Importantly, combinational therapies allow lower doses, hence less toxicity for healthy tissues and patients [95], currently achieved by simultaneously inhibiting two highly specific SL targets [96]. Table 1 shows some examples of SL CRISPR screens.

### 3.2. Synthetic Viable Screens

On many occasions, CRISPR screen approaches simultaneously search synthetic viability/resistance and lethality mechanisms to chemotherapy. GI screens are acting bidirectionally in order to set candidate hits that render tumor cells more sensitive or more resistance to chemotherapeutical drugs. In order to discover GI that confer resistance to BET bromodomain inhibitors (BBDIs), Shu et al. [98] used CRISPRko H1 and H2 libraries [23] targeting 18,000 protein coding genes to screen control and JQ1 treated TNBC cells. They found that deletion of *BRD2*, Mediator proteins, *AXL* and *TEAD1* kinase pathways or G1-S transition promoters sensitized parental and resistant cells to JQ1, however depletion of the BAF chromatin remodelling complex or ubiquitination-related genes (*SPOP*, *UBE2M*, *CUL3* and *USP14*) increased their resistance. To clarify resistance pathways to PARPi, Dev et al. [64] screened BRCA1 deficient breast cancer cell lines with the GeCKO library [45] and concluded that SHLD1/2 decreased expression confers PARPi resistance.

### 3.3. Novel Drug Targets Screens

Depending on the purpose of each study, the selected cells and drug concentrations will set the type of experiment to be conducted. Very sensitive cells to the selected drug allow screen stringent conditions, i.e., low treatment concentrations, to find the most essential GI with the therapy. However, a cell line more resistant to the treatment will require higher drug concentrations and will result in increased number of candidate hits, many of which would have minor phenotypes. Moreover, positive selection screens to identify gene hits that confer drug resistances, commonly have a high signal-to noise ratio, since only mutated resistance genes survive; on the contrary, in negative selection screens cells grow for ten or more population doublings to induce sensitization of cells by mutations that confer moderate phenotypical deficiencies [99]. Due to the complexity of chemical treatments in divergent genetic backgrounds like in cancer cell lines, Colic et al. [99] developed the drugZ algorithm to improve analysis of CRISPR screens that more precisely identifies GI to determine drug molecular mechanisms, treatment susceptibilities and resistances, and new drug targets.

The major obstacle for cancer therapy is cancer recurrence. Combined drugs offer good potential to circumvent not only cancer reappearance, but also lessen the doses and their inherent toxicity. At this aim, drug repurposing is central for the simplicity and promptness that could reach patients, although new therapies are discovered, there is a drug productivity gap [95]. A compendium of integrative information including 941 genome-wide CRISPR screens, performed in a human cancer cell panel from 30 cancer types, was designed to find potential therapeutic targets applied to patient selection in clinical studies [100]. In addition, 31 CRISPR screens including 27 genotoxic agents, to cover the vast majority of DNA damage types, screened with TKOv2 and TKOv3 libraries [37] into the p53 ko RPE1-hTERT cell line, determined 890 genes that when lost cause sensitivity or resistance to DNA damaging drugs [101]. Generating a genetic map of responses to genotoxic agents, authors identified particular roles in DNA repair pathways: ERCC6L2 in NHEJ pathway, ELOF1 in response to transcription-blocking drugs or the G-quadruplex ligand pyridostatin traps topoisomerase II on DNA. Multiple laboratories are working in finding out potential combinatorial therapies, some examples are listed in Table 2.

### 3.4. Pooled CRISPR Screens Based on FACS

Pooled CRISPR genetic forward screens based on FACS readouts offer an alternative way to conduct pooled CRISPR screens. They focus on protein levels determined by measuring the fluorescence intensity in response to drug, ligand or any regulator treatment. Pusapati et al. [106] used this technology to identify both positive and negative regulatory mechanisms in Hedgehog (Hh) signaling. Positive regulators comprehend *Rab34*, *Pdcl*, and *Tubd1* and are involved in ciliary functions and negative regulators include *Megf8*, *Mgrn1* and *Atthog*, which converged on the oncoprotein SMO. They constructed a clonal NIH/3T3 cell line with a GFP driven Hh-responsive promoter element, the fluorescence of which increased in response to the ligand Sonic Hedgehog (SHH) in a dose-dependent manner, thus providing a quantitative readout used for a genome-wide pooled screen based on FACS. They transduced the Brie library [39] into the engineered cells and treated them with high concentration of SHH for the positive regulators screens hence selecting candidate cells with lowest fluorescence; conversely, for the negative regulators screens, treatment was performed at low concentration of SHH and selected cells with high fluorescence. In another study, Mendelsohn et al. [107] integrate a pooled CRISPRi library targeting 2200 genes enriched with mitochondrial targets with a fluorescent biosensor and used fluorescence resonance energy transfer (FRET) and FACS to screen a metabolite at real-time level. These authors identified mitochondrial ribosomal proteins as essential to maintain energy levels and cell growth.

Furthermore, Jaitin et al. [108] propose the CRISPR-seq methodology to unravel complexity of biological circuits, combining massively parallel single-cell RNA sequencing (scRNA-seq) that enables further resolution to characterize regulation of cellular processes (and allows reduced false positive hits) with pooled CRISPR screens. The lentiviral vector used for sgRNA expression included a transcribed poly-adenylated unique guide index (UGI) to identify the sgRNA from scRNA-seq data and a fluorescent marker for studying perturbed cells in vivo. These authors used FACS to perform their screen according to the expression of two other lentiviruses sgRNA(CD11b)-BFP-UGI and sgRNA(Cebpb)-mCherry-UGI, to analyze modulation of immune and inflammatory responses, indeed, a revolutionary technique to elucidate the many mechanisms of cancer regulation.

### 3.5. 3D Cultured Cancer Models CRISPR Screens

Multicellular spheroid 3D models have been defined to more closely reproduce in vivo tumor conditions, compared to 2D cultured cells. They have been proven to better resemble cellular growth, cell-to-cell communication, gene expression or signal transduction in vivo, and therefore, they offer a more faithful environment for disease modeling or drug screening, reviewed in [109]. 3D cultured organoids recapitulate organ physiological parameters and, as they may be derived from stem cells, these cultures can self-renew and differentiate. Some laboratories have successfully established their own protocols to perform forward genetic CRISPR screens in valuable 3D cultured models: 3D spheroids [110,111] and 3D organoids [112,113,114,115], despite sgRNA heterogeneous efficiency due to spontaneous intrinsic differentiation or high false positive rates [115] and challenging genome-wide screen scaling [114]. These authors support the use of CRISPR screens in 3D cultured cancer models for the identification of gene functions to determine tumorigenesis, disease progression, novel drug targets and reduce or even replace animal models. CRISPR screens in 3D cultured cancer models are summarized in Table 3.

### 3.6. Ex Vivo and In Vivo CRISPR Screens

The ex vivo screens are performed in cultured primary cell lines that have been extracted from living animals. Cortez et al. [116] developed a pooled CRISPR screen platform in primary mouse T regulatory (Tregs) cells to find regulators of autoimmunity and anti-tumor immune responses. They designed a ~490 nuclear factors sgRNA library and used retroviral vectors to transduce into Tregs ex vivo. After staining Fox3p protein, authors determined the highest and lowest Foxp3-expressing cells and identified Usp22 and Atxn7l3 as positive, and Rnf20 as negative regulators.

The in vivo screens consist on transducing cells in vitro and subsequently transplanting them into animals in order to complete the screen. The number of cells to be implanted and the engraftment efficiency may restrict the experiment. Several laboratories have already implemented this approach; a summary is shown in Table 4.

In vivo screens may also be conducted by transduction of tissues in vivo, however, the accessibility and cell number to infect is restricted, and hence screens are limited to subpooled libraries. As an example, Hong et al. [124] investigated key roles of microRNAs in lung cancer. They engineered a dual guide RNA (dgRNA) system targeting each 16 miRNA precursor to screen for tumor-suppressive miRNAs in *KP* mouse model by lentiviral particles inhalation. The authors validated miR30b, miR146a and miR-190b as tumor suppressors.

### 3.7. Non-Coding Gene Targets CRISPR Screens

98% of our genomes contains non-coding DNA regions, where many oncogenic mutations may occur and whose biological function is still unknown [125]. Besides, most of these regions comprehend regulatory elements or are actively transcribed to RNA [126]. Therefore, dysregulation of these sequences are frequent in cancers [127]. CRISPR screens are emerging as a powerful toolkit to decode millions of potential regulatory elements for the ~20,000 translated genes. Previous numerous efforts to screen for enhancers using the CRISPR technology were restricted to mini-libraries [128,129,130,131,132,133,134,135,136], but recently, Gasperini et al. [137] combined scRNA-seq to detect gene expression changes with high MOI transduced sgRNAs for introducing multiple perturbations per cell. They transduced the chronic myelogenous leukemia K562 cell line at high MOI, with a 5920 paired sgRNA targeting candidate enhancer CRISPRi library and achieved around 28 multiplexed CRISPRi perturbations per single-cell transcriptome that allowed them to identify 664 *cis* enhancer-gene pairs.

Moreover, long non-coding RNAs (lncRNAs) are transcribed from thousands of *loci* in our genome and are involved in different cellular processes like transcriptional regulation, cellular reprogramming or differentiation, and also, they have been implicated in human diseases, such as cancer [138]. LncRNAs display specific cell-type expression and function [139], which reinforces the relevance of defining lncRNAs dysfunctions in tumor development and potential roles in therapy responses. LncRNAs function in *trans* or in *cis*. *Cis*-acting lncRNAs regulate gene expression depending on their own sites of transcription, at diverse distances of gene targets by activating, repressing or modulating their expression, reviewed in [140]. Consequently, it is highly important to discern the biological activities of lncRNAs, since they are major components of the mammalian genome. Actually, in the recent years, pioneer laboratories developed sophisticated methodologies to screen for lncRNAs cancer mechanisms. Interestingly, Liu et al. [141] screened 5689 lncRNA target CRISPRi library (the lncRNAs expressed in U87 cell line, derived from the CRiNCL library [142]) in the human glioblastoma cells and identified 33 hits that sensitize cells to radiotherapy and using human brain organoids they validated *lncGRS-1* as a novel glioma-specific therapeutic target enhancing radiotherapy. In another CRISPRa screen for the responses of melanoma cells to the BRAF inhibitor vemurafenib, Joung et al. [143] transduced a 10,504 intergenic lncRNA targeting TSSs sgRNAs into A375 cells and found 11 novel lncRNAs that mediate resistance to vemurafenib and further characterized that transcriptional activation of EMICERI activates the expression of four neighboring protein-coding genes, being any of them sufficient to confer BRAF inhibitor resistance. In another study with the aim of publishing a database of protein coding and lncRNAs involved in chemotherapy resistance to cytarabine, Bester et al. [144] developed a dual protein-coding and non-coding CRISPRa screening platform. They analyzed transcriptome data from 760 cancer cell lines to correlate effects of lncRNAs with phenotypes and used both a 14,701 lncRNA target library and a protein-doing library to transduce acute myelogenous leukemia cell lines. Authors found that transcriptional activation of GAS6-As2 lncRNA hyperactivates a resistance mechanism in multiple cancers via the GAS6/TAM pathway. Finally, to demonstrate that genome-wide screening of lncRNA function in site splicing (by exon skipping or intron retention), Liu et al. [145] determined cell-type-specific differences caused by lncRNAs by transducing a 10,996 lncRNA targetting library into: the K562 cell line (and found 230 lncRNAs essential for growth), GM12878 lumphoblastoid cells and HeLa cells.

Additionally, microRNAs (miRNAs) regulate gene expression post-transcriptionally and regulate tumorigenesis, metastasis and proliferation [146]. CRISPR screens using the GeCKOv2 library [45] that include 1864 sgRNAs against miRNAs already recognized the contribution of miR-152 and miR-345 in lung cancer metastasis [117] and miR-155 in myeloid leukemia cell proliferation [147]. However, Kurata and Lin [148] constructed an optimized on-target activity 1594 miRNA target library against the 85% of annotated human miRNA stem-loops and used it to define potential oncogenic and tumor suppressor miRNAs in cervical and gastric cancer cell lines. They successfully identify novel miRNAs by combining their results with expression and dysregulation of clinical data.

## 4. Future Perspectives

CRISPR screen technologies are accelerating cancer research field by illuminating the molecular mechanisms of tumorigenesis and chemotherapeutical investigation. sgRNA delivery, specificity and efficiency depend on genetic context and cellular type, being highly advisable to use more than one cell line for the screen and candidate genes validation. Despite these limitations, recent improvements in library design and analysis tools, together with the multitude of CRISPRko screens successfully completed have generated valued data that have already been integrated computationally in accessible interfaces, with the aim of creating the resources to match the genomics of cancer with a suitable treatment for each individual patient.

Moreover, CRISPR/Cas genetic screens are lately expanding to primary cells, 3D cultures (spheroids and organoids) or in vivo. All these cancer models offer a more accurate microenvironment to investigate tumorigenesis, disease progress or drug responses that enable valuable and sensitive novel GI discoveries. Nevertheless, in these cases sgRNA delivery, lentiviral transduction, library representation and coverage or experimental replications may be still challenging.

Regarding cancer immunotherapy, significant advances in tumor antigen presentation, tumor-specific T cells activity or tumor immune-induced cytotoxicity sensitization are already achieved by CRISPR screens strategies, reviewed in [149,150].

Future applications of other endonucleases as Cas12a that enables multiplexing of sgRNAs [73], and Cas13 that directly targets and edits cytoplasmic and nuclear RNAs [151,152] may soon change the strategy for SLI screens and transient in vitro and in vivo CRISPR screens. Moreover, the discovery of new gene families with divergent roles to adaptive immunity, opens up multiple possibilities to engineer cells at distinct levels (signal transduction, regulatory functions and others) [12].

Interestingly, CRISPRko screens are limited to less than 2% of our genome, as functional exons are a small part of our entire genome. Therefore, emerging screening analysis with CRISPRi and CRISPRa are recently providing extremely powerful information about differential regulators of essential genes and place the importance of non-coding regions as the new focus of researchers’ attention. These already present CRISPR applications are vastly crucial for novel diagnosis and therapeutical strategies.

Finally, CRISPR screens may be combined with scRNA-seq [108,137], protein barcodes [153] or real time monitoring procedures [107,154] to elucidate highly complex regulatory pathways integrating single cell resolution, in situ conditions or real dynamic detection.

The growing explosion of these methodologies are currently remodeling oncology research by redefining cancer heterogeneity, biomarkers and therapies, thus gradually resolving the existing limitations to become excellent and routine tools in the close future.

## Figures and Tables

**Table 1:** Synthetic lethal CRISPR screens.

System	Library	Model	Analysis	SLI between
CRISPR-based double KO [97]	21,321 pairs of drug targets	K562 leukemia cells	casTLE	DNA repair proteins APEX1 and ATM, anti-apoptotic BCL2L1 and MCL1
CRISPR-based double KO [62]	119 KRAS interactor targets	lung adenocarcinoma cells	UPGMA scipy statsmodels	RAS adhesion controller RADIL and endocytosis regulator RIN1, *RAP1GDS1* and *RHOA*
Enhance alisertib Aurora-A inhibitor activity [69]	507 kinase targets	Breast cancer cells	MAGeCK	GSG2 inhibition (interfering with AURORA-B) significantly decreased tumor growth in vitro and in vivo

**Table 2:** Novel drug targets CRISPR screens.

Aim	Library	Model	Treatment	Analysis	Targets
Resistances to FLT3 inhibitor [102]	GeCKO	MV4-11 acute myeloid leukemia cells	Quizartinib	Self-calculated	Expression of SPRY3 and GSK3A was significantly decreased in resistant cells
Resistances to multi-targeted tyrosine kinase inhibitors (TKIs) [70]	Customized library (18,000 targets)	clear cell renal cell carcinoma (ccRCC)	Sunitinib	Self-calculated	farnesyltransferase expression as a factor of sunitinib resistance
Asparaginase responses [63]	GeCKO	acute leukemia cells (ALC)	Asparaginase	MAGeCK v0.5.7	Wnt signalling induced asparaginase sensitivity in resistant ALC
Synergizes with metformin [66]	Brunello	U251 cells	Metformin	MAGeCK	Metformin and CDK4/6 inhibitor combination as tumoral therapy
Abemaciclib resistance CRISPR and CRISPRi screen [103]	Brunello and CRISPRi-v2	Hedgehog associated medulloblastoma cells	Abemaciclib	MAGeCK -VSIPR	Hedgehog signaling in neuroblastoma depends on smoothened-activating sterol lipids
Molecular pathways depending on ataxia-telangiectasia and Rad3-related (ATR) kinase [104]	TKOv1 and TKOv3	colon carcinoma HCT116, HeLa and a p53-mutated clone of RPE1 hTERT cells	ATR inhibitors VE-821 and AZD6738	MAGeCK and drugZ	DNA replication, DNA repair and cell cycle regulators give hypersensitive to ATR inhibitors. POLE3/POLE4 proteins are potential biomarkers for ATR processes.
Druggable targets in RNF43-mutant pancreatic adenocarcinomas [67]	TKO gRNA library	HPAF-II human pancreatic ductal adenocarcinoma cell line	-	BAGEL algorithm	Wnt receptor Frizzled-5 (FZD5)
Druggable targets in *Keap1a*-mutant or NRF2-hyperactive tumors [105]	4,915 druggable targets library	Murine lung adenocarcinoma (LUAD) Kras^G12D/+^; p53^−/−^ (KP) versus Kras^G12D/+^; p53^−/−^; Keap1^−/−^ (KPK) cell lines	-	RSEM, JADE algorithm and GSEA	SLC33A1 and unfolded protein response related genes are novel targets for patients harboring *KEAP1*-mutant or NRF2-hyperactivated tumors

**Table 3:** 3D cultured cancer model CRISPR screens.

Aim	Library	Model	Method	Analysis	Targets
Cancer biomarker and therapy [110]	Customized CRISPR ko library	H23 LUAD 2D cell line and 3D spheroids	3D versus 2D cultures	computed t-value scores	p53 and Ras are 3D hits, IGF1R expression/dependency and KRAS mutation may serve as biomarkers
NRF2 hyperactivation-induced spheroid growth and NRF2-hyperactivated tumors [111]	Customized 1,500 NRF2-hyperactivated related gene targets library	A549 and H1437 LUAD 2D cell line and 3D spheroids	3D versus 2D cultures	MAGeCK-VISPR	In spheroids, loss of TSC1 enhances inner clearance and depletion of GPX4 enhances proliferation
Resistances to TGF-β-mediated growth restriction [114]	283 potential tumor suppressor genes customized library and the Brunello library	Human small intestinal (hSI) organoids	wild-type versus APC mutant and APC and TP53 double mutant human intestinal organoids	MAGeCK V0.5.4	Multiple subunits of the tumor-suppressive SWI/SNF chromatin remodeling complex
Tumor drivers in colorectal cancer (CRC) [115]	85 tumor suppressor genes customized library	Pre-malignant organoids with APC^−/−^; KRAS^G12D^ mutations	Primary organoids versus cancer cell lines	CRISPR- ERA	TGFBR2 and CRC growth mediators

**Table 4:** In vivo CRISPR screens.

Aim	Library	Model	Method	Analysis	Targets
Loss-of-function screen in tumor growth and metastasis [117]	mGeCKOa	Tumor-inducible non-small-cell lung cancer (NSCLC) cell line	Metastasis versus primary tumors	GSEA	Mutations that inactivate apoptosis and Nf2, Trim72, Ptges2 genes in primary tumor cells, and Ube2g2 mutations in metastasis
Epigenetic regulators of tumor immunity [118]	Customized epigenetic sgRNA subpooled	Murine Kras^G12D^/Trp53^−/−^ LUAD	Anti-PD-1 or isotype control treatments	DESeq2, MAGeCK and GSEA	Asf1a reduced in tumors of WT mice treated with anti–PD-1
Cellular composition and architecture of cutaneous squamous cell carcinoma (cSCC) [119]	Low expressed xenograft genes subpooled	Several cSCC cell lines	Tumor analysis	STARS	TSK-enriched integrin signaling genes *ITGB1*, *FERMT1* and *CD151*.
Non-small cell lung cancers treatment [68]	Customized epigenetic sgRNA subpooled	KP, non-small cell lung cancers cells	Tumor analysis	GSEA, MSigDB and DESeq2	Npm1
Synergistic lethal drug interactions with MEK signalling pathway inhibitors to treat pancreatic ductal adenocarcinoma (PDAC) [120]	Nuclear subpooled [15]	PDX366 cells from pancreatic patients	Trametinib treatment	DREBIC	CENPE and RRM1 inhibition are sensitizers to trametinib
SL combinatorial target with gemcitabine to treat PDAC [121]	Customized epigenetic sgRNA subpooled	PDX366 cells from pancreatic patients	Gemcitabine treatment	MAGeCK v0.5.2.	Inhibition of PRMT5 increases cytotoxicity to gemcitabine
Find mechanisms of resistance to docetaxel to treat metastatic prostate cancer [122]	GeCKOv2A	deficient Pten and Spry2 model cells	Docetaxel treatment	MAGeCK v0.5.6.	Suppression of TCEAL1 enhances tumor sensitivity to docetaxel
Gene activation screen in vivo [123]	CRISPRa sgRNA targeting 25 DNA damage regulators	dCas9-VP64-expressing Bcr-Abl– driven murine acute B-cell lymphoblastic leukemia cells	Temozolomide treatment	DESeq and self- calculated	Transcriptional activation of tumor suppressor Chek2 sensitizes tumor cells to temozolomide

## Data Availability

The data presented in this study are available on supplementary (Table S1) information.

## Supplementary Materials

The following are available online at https://www.mdpi.com/article/10.3390/cancers13071591/s1, Table S1: Data presented in this study.
